# Satisfaction with ophthalmology residency training from the perspective of recent graduates: a cross-sectional study

**DOI:** 10.1186/1472-6920-13-75

**Published:** 2013-05-27

**Authors:** Tatiana Millán, Keila Monteiro de Carvalho

**Affiliations:** 1 Department of Ophthalmology, State University of Campinas, Campinas, São Paulo, Brazil

**Keywords:** Medical education, Medical residency program, Ophthalmology

## Abstract

**Background:**

Few studies have evaluated satisfaction with medical residency programs from the perspective of residents or recent graduates. Knowledge of current conditions of teaching might help to identify deficiencies and to provide adequate training. So, the aim of this study was to assess the satisfaction with residency training and to identify deficiencies in this training from the perspective of recent graduates in ophthalmology residency.

**Methods:**

For this purpose, we developed a questionnaire and gaved it to recent graduates in ophthalmology residency in São Paulo, Brazil, from January to December 2010. The questions contained demographic information (age, sex and time of practice in ophthalmology), a Likert scale to evaluate the level of satisfaction with medical residency concerning clinical knowledge, surgical skills and doctor-patient relationship and questions about deficiency in clinical and surgical areas.

**Results:**

The areas in which recent residency graduates were very or extremely satisfied were: acquisition of clinical knowledge (89.1%), acquisition of surgical skills (93.4%) and the development of doctor-patient relationship (74.9%). Specific areas of clinical knowledge in which they perceived more deficiency were orbit (48.3%) and ophthalmic pathology (47.9%), and in surgical skills were refractive surgery (65.9%) and orbit (59.2%)

**Conclusions:**

The assessment of the satisfaction with residency training in ophthalmology from the perspective of recent graduates showed high level of satisfaction and identified specific deficiencies in ophthalmic pathology, refractive surgery and orbit.

## Background

The goal of medical residency is the improvement of the medical practice indifferent areas. [1]. The National Committee of Medical Residency that accredits medical residency programs in Brazil established that 80% to 90% of the time of medical residency should be developed in the form of in-service training with the theoretical activities in the remaining time [2]. In ophthalmology residency, teaching includes clinic training, acquisition of surgical skills and development of doctor-patient relationship [3].

Several studies have evaluated medical residency in many areas of medicine, such as psychiatry, gastroenterology, neurology, anesthesiology and other specialties. A study in pediatric residency evaluated how the practice contributes to developing clinical reasoning and diagnostic and therapeutic techniques [4]. Another study examined twenty-six residency training programs in gastroenterology, accredited by National Committee of Medical Residency in Brazil, through open questionnaires to identify the number and the qualifications of preceptors of programs, the frequency of journal club, the number of hours attending outpatient clinics, the access to more complex treatment and the processes of assessment of medical residents [5]. Psychiatry residency training was evaluated through site visits, semi-structured interviews and questionnaires applied to samples of residents and preceptors [6]. Another study evaluated difficulties in patient care and sources of stress during medical residency training [7]. Oliveira-Filho [8] studied the acquisition of knowledge, learning strategies, satisfaction with learning environment and quality of life of residents in anesthesiology. None of these studies evaluated the satisfaction with residency training from the perspective of residents.

In ophthalmology we identified some studies in this area. Melo identified the qualifications of ophthalmic educators from a university hospital in Recife, Brazil, using open-ended and closed-ended questioning [9]. In another study that evaluated postgraduate ophthalmic education in the United States of America, 83% of the doctors who had attended the courses in ophthalmology felt satisfied [10]. Abramoff et al. reviewed the pertinent literature on education in retinal lasers in ophthalmology residency and concluded that there was no standardized curriculum for teaching and assessing resident competencies in retinal lasers [11]. In a systematic literature review, it was suggested the use of a laboratory to teach and assess aspects of resident surgical competence [12]. In another study, specific techniques for teaching and assessing surgical competence in ophthalmology residencies were suggested, such as: written and explicit goals or objectives for each stage of training and the use of formative rather than summative feedback [13]. Golnik et al. developed a 1-page on-call assessment tool (OCAT) and scoring rubric to evaluate ophthalmology resident on-call performance. A retrospective chart audit of consecutive resident on-call charts was performed using the OCAT and they concluded that it could be used to assess resident competence in patient care, professionalism and medical knowledge [14].

The ophthalmology training programs in Brazil are evaluated only by objective parameters such as the number of hours dedicated to outpatient clinic and emergency rooms, the number of surgeries performed by each medical resident, the frequency of lectures and other learning experiences. They are not evaluated from the perspective of residents. Some residency training programs perform theoretical and practical assessments of their residents, but generally the opinions of the residents are not considered. Most studies have been conducted with the objective of assessing the skills acquired by the residents during ophthalmology residency programs using objective parameters, but few studies have evaluated the satisfaction with ophthalmology residency according to the residents’ perceptions. Accordingly, the aim of this study was to assess the satisfaction with residency training and to identify deficiencies in this training from the perspective of recent graduates in ophthalmology residency.

## Methods/design

Cross-sectional study.

### Survey instrument

We designed a questionnaire to assess satisfaction with residency training in ophthalmology and to identify deficiencies in the training of clinical and surgical areas. The areas, clinical and surgical, were based on the Mandatory Training Units for accreditation by the Brazilian Council of Ophthalmology, specified in Table 1. Additional file 1: Table S1 shows the applied questionnaire.

**Table 1:** Mandatory training

**Mandatory training**	**Percentage of minimum hours (%)**
Oculoplastic	5
Neuro-ophthalmology	5
Refractive Surgery	5
Prevention of Blindness and Visual Rehabilitation	5
Orbit	5
External Disease/Cornea	10
Strabismus and Pediatric Ophthalmology	10
Uveitis	5
Ophthalmic Pathology	5
Emergency	10
Glaucoma	10
Optics/Refraction and Contact Lenses	15
Retina	10

### Sample

Inclusion criteria were:

[1] Graduation in an ophthalmology residency in the state of São Paulo, Brazil, accredited by the National Committee of Medical Residency and by the Brazilian Council of Ophthalmology in the last five years;

[2] a 36-month period of residency training.

We opted to study the residents of the state of São Paulo because almost fifty percent of the ophthalmology residents in Brazil have their training in this state, in twenty-six Ophthalmology training programs.

Four hundred and thirty five physicians met these criteria. The true prevalence of ophthalmologists that considered their residency satisfactory in Brazil was unknown because there are no studies that have evaluated this question. Accordingly, a hypothetic proportion of 50% was assumed for the calculation of the sample size with a 95 % confidence interval. The calculated sample size with a 95% confidence interval was of 211 subjects.

### Statistical analysis

Pearson's Correlation Coefficient was used to measure the degree of association between the following variables: acquisition of clinical knowledge, acquisition of surgical skills and development of the doctor-patient relationship. The Odds Ratio Chi-Square Test was used to determine whether there was association and the degree of association between perception of deficiency in a specific clinical area and its correlated surgical area.

### Ethics

This study was approved by the Research Ethics Committee of the Faculty of Medical Sciences of State University of Campinas, SP, Brazil, which was accepted as institutional review board by the Universal Declaration on Bioethics and Human Rights adopted by United Nations Educational Scientific and Cultural Organization.

## Results

Two hundred and eleven recent graduates answered the questionnaire. Ages ranged from 26 to 40 years old: 88 (41,7%) were 26 to 30 years old, 113 (53.6%) from 31 to 35 years old and 10 (4.7%) were 36 to 40 years old. Ninety-five (45%) were men and 116 (55%) were women.

### Overall level of satisfaction

Table 2 shows the level of satisfaction with medical residency in acquisition of clinical knowledge, acquisition of surgical skills and development of the doctor-patient relationship.

**Table 2:** Level of satisfaction with medical residency

	**Clinical knowledge (%)**	**Surgical skills (%)**	**Doctor-patient relationship (%)**
Not at all satisfied	4 (1.9%)	1 (0.5%)	3 (1.4%)
Slightly satisfied	3 (1.4%)	1 (0.5%)	7 (3.3%)
Moderately satisfaction	16 (7.6%)	12 (5.7%)	43 (20.4%)
Very satisfied	102 (48.3%)	101 (47.9%)	89 (42.2%)
Extremely satisfied	86 (40.8%)	96 (45.5%)	69 (32.7%)
Total	211 (100%)	211 (100%)	211 (100%)

There was a moderate positive correlation between the level of satisfaction with acquisition of clinical knowledge and the level of satisfaction with development of the doctor-patient relationship (Pearson Correlation r=0.497, p<0.001), a weak positive correlation between the level of satisfaction with acquisition of surgical skills and the development of the doctor-patient relationship (Pearson Correlation r=0.307, p<0.001) and a weak positive correlation between the level of satisfaction with acquisition of clinical knowledge and with acquisition of surgical skills (Pearson Correlation r=0.219, p=0.001).

### Clinical knowledge – specific areas

Analysis of deficiency in specific areas of clinical knowledge showed that the areas that were perceived as deficient were orbit (48.3% of the subjects marked it) and ophthalmic pathology (47.9%). The areas perceived as less deficient were glaucoma (2.4%) and ocular emergencies (4.3%). Table 3 shows all percentages.

**Table 3:** Deficiency in clinical knowledge areas-overall percentages

	**No deficiency (%)**	**Deficiency (%)**
Oculoplastic	85.3	14.7
Neuro-ophthalmology	75.4	24.6
Prevention of blindness and visual rehabilitation	68.7	31.3
Orbit	51.7	48.3
External Disease/Cornea	82.9	17.1
Strabismus	74.9	25.1
Pediatric Ophthalmology	83.4	16.6
Uveitis	94.3	5.7
Ophthalmic Pathology	52.1	47.9
Emergency	95.7	4.3
Glaucoma	97.6	2.4
Optics/Refraction	85.8	14.2
Contact Lenses	89.1	10.9
Retina	89.1	10.9

### Surgical skills - specific areas

Analysis of deficiency in surgical skills in each specific ophthalmic surgical areas showed that areas perceived as more deficient were refractive surgery (65.9% of the subjects perceived deficiency) and orbit (59.2%). Areas perceived as less deficient were cataract surgery (5.7%), glaucoma (8.1%) and ocular trauma (4.3%). Table 4 shows all percentages.

**Table 4:** Deficiency in surgical skills by area – overall percentages

	**No deficiency (%)**	**Deficiency (%)**
Plastics/Reconstructive surgery	75.8	24.2
Refractive Surgery	34.1	65.9
Orbit Surgery	40.8	59.2
External Disease/Cornea Surgery	80.1	19.9
Cataract Surgery	94.3	5.7
Strabismus Surgery	75.8	24.2
Ocular Trauma Surgery	88.6	11.4
Glaucoma	91.9	8.1
Retina/Vitreous surgery	57.8	42.2

### Correlation areas of clinical knowledge/surgical areas

The areas correlated were: oculoplastics clinical knowledge/plastics/ reconstructive surgery, optics/refraction clinical knowledge/refractive surgery, orbit clinical knowledge/orbit surgery, external disease and cornea clinical knowledge/external disease and corneal surgery, strabismus clinical knowledge/strabismus surgery, emergency clinical knowledge/ocular trauma surgery, glaucoma knowledge/glaucoma surgery and retina clinical knowledge/retina and vitreous surgery.

Table 5 shows that odds ratio (OR) for perceiving deficiency in some specific surgical area increased significantly when subjects had perceived deficiency in clinical knowledge of correlated area, except for optics and refraction/refractive surgery where the association was not significant.

**Table 5:** Odds ratio for deficiency in surgical skills according to deficiency in clinical knowledge

**Area of Clinical Knowledge/Area of Ophthalmic Surgery**	***p *value**	**OR**	**95% CI**
Oculoplastic/Plastics/Reconstructive Surgery	<0.001*	227.14	29.42-1753.3
Optics and Refraction/Refractive Surgery	0.25	1.84	0.75-4.52
Orbit/Orbit Surgery	<0.001*	4.7	2.58-8.57
External Disease and Cornea/External Disease and Corneal Surgery	<0.001*	17.44	7.47-40.7
Strabismus/Strabismus Surgery	<0.001*	50.56	20.4-125.33
Emergency/ Ocular Trauma Surgery	0.001*	13.04	2.97-48.68
Glaucoma/Glaucoma Surgery	0.003*	13.35	1.76-101.2
Retina/Retina and Vitreous Surgery.	<0.001*	39.73	5.24-301.34

*OR *odds ratio, *CI *confidence level, * significant difference.

## Discussion

The results show that the level of satisfaction from the perspective of recent graduates of Ophthalmology residency training was high: 89.1% were very satisfied or extremely satisfied with the acquisition of clinical knowledge and 93.4% were very satisfied or extremely satisfied with the acquisition of surgical skills. Only one study [10] has looked at the satisfaction with the post graduation in ophthalmology (83%), and this showed results similar to ours findings.

We found a moderate positive correlation between the level of satisfaction with acquisition of clinical knowledge and the development of the doctor-patient relationship, and weak positive correlations between the level of satisfaction with acquisition of surgical skills and the development of the doctor-patient relationship, as well as between the level of satisfaction with acquisition of clinical knowledge and the acquisition of surgical skills. This is probably because the subjects that were more satisfied perceived more satisfaction in all aspects.

Despite the satisfaction with the acquisition of clinical knowledge being high, the subjects identified some deficient areas, especially orbit (48.3%) and ophthalmic pathology (47.9%). This is probably because these areas are not traditionally emphasized in ophthalmology residency programs in Brazil, despite the recommendations of the Brazilian Council of Ophthalmology. There are few ophthalmologists in Brazil that have experience in orbit.

In the assessment of surgical skills, analysis showed that the areas that were perceived as more deficient were refractive surgery (65.9%) and orbit (59.2%). One possible reason for deficiency in orbit may be that in Brazil, few ophthalmologists perform orbital surgery and that many refer their patients to other professionals, such as oral and maxillofacial surgeons. However, the residency training programs evaluated in this study are the main centers in ophthalmology in Brazil, where the most complex surgeries are performed. That even graduates of these programs would feel deficient in orbital surgery would indicate that surgery should be more emphasized in residency training. In the case of refractive surgery, another perceived area of deficiency, only one residency program in Brazil that was evaluated in this study has training in this type of surgery, even though this is a frequently performed surgery in Brazil. Besides that, the training in refractive surgery is mandatory for the accreditation of residency training programs in Brazil.

Results also show the odds ratio (OR) for perceiving deficiency in some specific surgical area when it had been perceived deficiency in clinical knowledge. Because of the correlation between a specific clinical area and its respective surgical area, when the subjects had perceived that clinical knowledge was deficient, they also had perceived similar deficiency in the surgery. This is an expected result because the acquisition of surgical skills has to be preceded of clinical knowledge, especially in some areas such as oculoplastic (OR: 227.14) and strabismus (OR: 50.56). These are areas where the clinical knowledge is much correlated to the surgery. It just did not happen with the area of optics/refraction and refractive surgery because the teaching of this surgery was inadequate not due to lack of clinical knowledge, but due to lack of infrastructure for teaching this surgery.

We found that the satisfaction with the development of the doctor-patient relationship (74.9%) was not as high as satisfaction with clinical knowledge and surgical skills possibly because the evaluated residency programs are not working well or are not thorough enough in this area.

### Limitations of this type of study

All data collection instruments which use only the perceptions of the research subjects have high subjectivity, which may reflect more or less accurately the reality. Thus, perceptions of graduates may not be the best tool to objectively measure the quality of medical residencies, because at this point, objective examinations performed by Brazilian Council of Ophthalmology would be more appropriate. Nevertheless, the views and perceptions of graduates provide some useful information to assess whether the residency programs are preparing them adequately for the practice from their perspectives. The perceptions of graduates are rarely considered to assess residency programs and, to our knowledge, this is the first study on perceptions of graduates of medical residencies in ophthalmology in Brazil.

## Conclusions

In this study, the satisfaction with residency training in ophthalmology from the perspective of recent graduates in São Paulo, Brazil was high. Despite of that, the graduates identified deficiency specially in the following areas: ophthalmic pathology, orbit and refractive surgery. We should improve training in these areas in the evaluated residency programs.

## Competing interests

The authors declare that they have no competing interests.

## Authors' contributions

TM participated in the conception and design of the study, in the acquisition, analysis and interpretation of data and drafted the manuscript. KMM participated in the conception and design of the study, in the analysis and interpretation of data and coordinated and helped to draft the manuscript. All authors read and approved the final manuscript.

## Pre-publication history

The pre-publication history for this paper can be accessed here:

http://www.biomedcentral.com/1472-6920/13/75/prepub

## Supplementary Material

Additional file 1: Table S1 Questionnaire. Click here for file
