# Supplementary material for: Satisfaction with ophthalmology residency training from the perspective of recent graduates: a cross-sectional study
Source: BMC Med Educ. 2013 May 27;13:75. doi: 10.1186/1472-6920-13-75 (PMC3679975; doi:10.1186/1472-6920-13-75)
Supplement: Additional file 1: Table S1 — Questionnaire. [file 1472-6920-13-75-S1.doc]

Additional file 1

Table S1: Questionnaire

| 1) Please indicate your age:  ( ) until 25 years ( ) 26 to 30 years ( ) 31 to 35 years ( ) 36 to 40 years ( ) more than 40 years    2) Please indicate your gender: ( ) Male ( ) Female  3) How many years did you finish your residency training?  ( ) 1 year or less ( ) 2 years ( ) 3 years ( ) 4 years ( ) 5 years  4) Specifically thinking about your residency training program, how do you feel about the training in each of the areas below?   |  | Extremely  Satisfied | Very Satisfied | Moderately Satisfied | Slightly Satisfied | Not at all Satisfied | | --- | --- | --- | --- | --- | --- | | Clinical Knowledge |  |  |  |  |  | | Surgical Skills |  |  |  |  |  | | Doctor-patient Relationship |  |  |  |  |  |   5) After residency training, in what clinical areas, if any, did you not feel well prepared for practice?  ( ) Oculoplastics ( ) Neuro-ophthalmology  ( ) Prevention of blindeness and visual rehabilitation ( ) Orbit  ( ) External disease and Cornea ( ) Strabismus  ( ) Pediatric Ophthalmology ( ) Uveitis  ( ) Ophthalmic Pathology ( ) Emergency  ( ) Glaucoma ( ) Optics/Refraction  ( ) Contact Lenses ( ) Retina  6) After residency training, in what surgical areas, if any, did you not feel well prepared for practice?  ( ) Plastics and Reconstructive Surgery ( ) Refractive Surgery  ( ) Orbit Surgery ( ) External Disease and Cornea Surgery  ( ) Cataract Surgery ( ) Strabismus Surgery  ( ) Ocular Trauma Surgery ( ) Glaucoma Surgery  ( ) Retina and Vitreous Surgery |
| --- | --- | --- | --- | --- | --- | --- | --- | --- | --- | --- | --- | --- | --- | --- | --- | --- | --- | --- | --- | --- | --- | --- | --- | --- |
